# Donor Plasmid Optimization Enhances Expression of Feline Parvovirus VP2 Protein in the Baculovirus Expression Vector System

**DOI:** 10.3390/vaccines14010077

**Published:** 2026-01-10

**Authors:** Ziyan Meng, Zhen Sun, Jing Li, Wenjia Qiu, Jiaqi Wei, Ruitong Zhang, Xiaoyu Ji, Hongwei Zhu, Jiayu Yu, Yang Liu, Linlin Jiang, Jianlong Zhang, Xin Yu, Xingxiao Zhang

**Affiliations:** 1 School of Life Sciences, Ludong University, Yantai 264025, China; zymeng@migroupedu.cn (Z.M.); sunzhenzgsd@outlook.com (Z.S.); jli@migroupedu.cn (J.L.); wjqiu@migroupedu.cn (W.Q.); jqwei@migroupedu.cn (J.W.); rtzhang@migroupedu.cn (R.Z.); xyji@migroupedu.cn (X.J.); hwzhu@ldu.edu.cn (H.Z.); jyu@migroupedu.cn (J.Y.); yliu@migroupedu.cn (Y.L.); linlinjiang@ldu.edu.cn (L.J.); zhangjianlong@ldu.edu.cn (J.Z.); 2 Yantai Key Laboratory of Animal Pathogenic Microbiology and Immunology, Yantai 264025, China

**Keywords:** feline panleukopenia virus, VP2, virus-like particles, baculovirus expression vector system, immunogenicity

## Abstract

Background: Feline panleukopenia virus (FPV) causes acute and frequently fatal disease in cats, underscoring the urgent need for safe, rapidly effective, and scalable vaccines. While virus-like particle (VLP) vaccines are inherently safe and immunogenic, their development is constrained by low yields of recombinant protein in insect cell expression systems. Methods: An optimized baculovirus expression vector system (BEVS) incorporating the hr1-p6.9-p10 transcriptional enhancer and the Ac-ie-01 anti-apoptotic gene was employed to enhance recombinant protein production. VP2 expression levels, viral titers, and hemagglutination activity were quantified using qPCR, SDS-PAGE/Western blotting, transmission electron microscopy (TEM), and functional assays. Immunogenicity and protective efficacy were assessed in both mice and cats through serological analysis, neutralizing antibody detection, and post-challenge clinical monitoring. Results: The optimized BEVS enhanced recombinant protein transcription by 1.5-fold, viral titers by 3.7-fold, and hemagglutination activity by 15-fold. The purified protein self-assembled into uniform 25 nm virus-like particles (VLPs). Immunization elicited earlier responses compared to commercial vaccines. Vaccinated cats maintained normal body temperature, stable leukocyte counts, and minimal viral shedding following FPV challenge. Conclusions: This study validates an enhanced BEVS that effectively overcomes VP2 yield constraints and generates highly immunogenic FPV VLPs. The platform enables rapid-onset protection and offers a scalable strategy for next-generation FPV vaccine development.

## 1. Introduction

Feline panleukopenia virus (FPV) is an acute and highly contagious pathogen that causes severe systemic disease in felids, with mortality rates in young kittens reaching 50–90%. Vertical transmission via maternal infection can lead to fetal resorption, stillbirth, or cerebellar hypoplasia due to disrupted neurodevelopment [1]. The global endemicity of FPV enables its persistence in high-density settings such as animal shelters and breeding catteries, where outbreaks are particularly difficult to control [2].

FPV is a non-enveloped virus with a single-stranded DNA genome [3]. Its capsid is primarily composed of the structural protein VP2, which constitutes approximately 90% of the capsid mass and contains the major antigenic sites required for eliciting neutralizing antibodies [4]. Consequently, VP2 represents the most promising target for subunit vaccine development and serological diagnostics [5]. Although conventional inactivated or live-attenuated vaccines are effective, they carry biosafety risks associated with infectious viral material and typically require strict cold-chain storage [6]. Recombinant subunit vaccines or virus-like particle (VLP) vaccines offer enhanced safety and precise antigenic presentation, but their practical application is limited by low expression levels and inefficient assembly into structurally authentic capsids [7,8,9].

To overcome these limitations, multiple heterologous expression platforms have been explored, including Escherichia coli, mammalian cells, and the baculovirus expression vector system (BEVS) [10,11,12]. Bacterial systems allow rapid and cost-effective production, but frequently yield insoluble or misfolded VP2 lacking conformational epitopes [13]. Mammalian systems can provide authentic post-translational modifications, but are limited by high production costs and scalability [12,14]. In contrast, BEVS has emerged as a preferred approach for FPV VP2 and other viral capsid proteins due to its high-level expression, correct folding, and efficient VLP assembly [15].

Conventional BEVS platforms, such as the widely used Bac-to-Bac system, primarily rely on very late viral promoters (polh, p10) [16]. These promoters are activated during the late phase of infection when virus-induced apoptosis and cell lysis occur, limiting the effective expression window and reducing overall protein yield [17]. Previous attempts to enhance VP2 expression have largely focused on single-factor strategies, including promoter substitution, codon optimization, or host cell engineering [7,18]. While these approaches have achieved incremental improvements, they do not fully resolve the conflict between late promoter activation and declining cellular viability during infection [12].

Building upon these strategies, a dual-level optimization approach, integrating a hybrid hr1-p6.9-p10 regulatory cassette with the anti-apoptotic gene Ac-ie-01, has been shown to markedly enhance GFP production in insect cells [19]. This combined regulatory and anti-apoptotic strategy is expected to be more effective than single-factor modifications because it extends the productive expression window while maintaining cell viability. However, its effectiveness for high-yield expression of complex viral structural proteins, such as FPV VP2, and for efficient VLP assembly has not been systematically evaluated.

In this study, we employed the Bac-to-Bac platform in an optimized BEVS configuration, incorporating the coordinated regulatory and anti-apoptotic strategy to drive FPV VP2 expression. Comparative analyses under identical conditions demonstrate that the optimized BEVS significantly increases VP2 yield, supports VLP assembly, and elicits robust antibody responses in both murine and feline models. BALB/c mice were used to assess immunogenicity due to their well-characterized immune responses, while cats were included as the natural host to evaluate vaccine-induced protection, clinical outcomes, and viral shedding. Collectively, these findings establish a promising optimized BEVS platform for efficient VLP-based FPV vaccine development.

## 2. Materials and Methods

### 2.1. Viruses, Cells, and Reagents

The FPV strain FPV072 (GenBank accession no. OQ398406.1) [20], previously isolated in our laboratory, was used as the template for VP2 amplification. The pFastBac™ Dual vector and *E. coli* DH10Bac™ competent cells were obtained from Thermo Fisher Scientific (Waltham, MA, USA), and *E. coli* DH5α cells were purchased from Vazyme Biotech (Nanjing, China). Sf9 insect cells (Womei Bio, Suzhou, China) were maintained in serum-free Sf-900™ II SFM (Thermo Fisher Scientific) at 27 °C with constant agitation. Cellfectin^®^ II (Thermo Fisher Scientific) was used for bacmid transfection, and T4 DNA ligase (Vazyme Biotech) was used for plasmid construction. A monoclonal anti-parvovirus antibody (clone CPV1-2A1; Abcam, Cambridge, UK), originally raised against canine parvovirus (CPV), was used for FPV VP2 detection because CPV and FPV share highly conserved VP2 epitopes that allow reliable cross-reactivity. An HRP-conjugated anti-mouse IgG secondary antibody (Zhongshan Jinqiao, Zhongshan, China) was used for immunodetection.

### 2.2. Animals

Specific-pathogen-free (SPF) female BALB/c mice (6 weeks of age) and SPF British Shorthair cats (16 weeks of age) were obtained from licensed commercial vendors. All animals were confirmed seronegative for feline panleukopenia virus (FPV) prior to immunization. All animal procedures were approved by the Animal Ethical and Experimental Committee of Ludong University on 15 December 2023 (license number LDU-IACUC2023007) and conducted in accordance with the National Institutes of Health Guide for the Care and Use of Laboratory Animals. Animals were housed under specific pathogen-free (SPF) conditions with controlled temperature (22 ± 2 °C), a 12 h light/dark cycle, and ad libitum access to food and water.

### 2.3. Construction of the Optimized Donor Plasmid

To enhance VP2 expression, an optimized donor plasmid (pFastBac Dualopt, pFBDopt) was constructed by inserting the AcMNPV hr1 enhancer and p6.9 promoter upstream of the p10 promoter, forming a composite regulatory cassette (hr1-p6.9-p10) designed to drive early and sustained transcription. The anti-apoptotic gene Ac-ie-01 was placed under the control of the polh promoter to delay host cell apoptosis and extend the recombinant protein expression period. This synthetic expression cassette was cloned into the pFastBac Dual vector backbone to generate the optimized BEVS donor construct (Nanjing, China).

**Figure 1:**
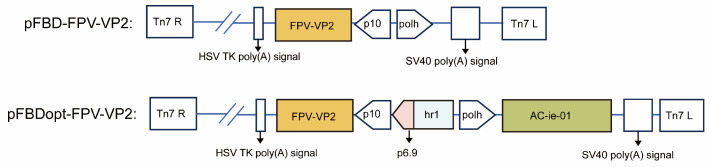
The VP2 gene was placed under the control of an hr1-p6.9-p10 composite promoter to enable early and sustained transcriptional activity, while the Ac-ie-01 anti-apoptotic gene was integrated into the construct to prolong host cell viability.

The FPV VP2 gene was amplified from the FPV072 strain using primers designed with SnapGene v6.0.2 and inserted into the conventional and optimized donor plasmids via HindIII/BamHI and KpnI/XhoI restriction sites, respectively. The resulting plasmids were designated pFBD-FPV-VP2 and pFBDopt-FPV-VP2. All recombinant plasmids were verified by colony PCR, restriction enzyme digestion, and Sanger sequencing at Sangon Biotech (Shanghai, China), confirming correct insertion and sequence integrity (Figure 1).

### 2.4. Generation of Recombinant Bacmids

The verified plasmids (pFBD-FPV-VP2 and pFBDopt-FPV-VP2) were transformed into *E. coli* DH10Bac cells to enable Tn7-mediated site-specific integration of the VP2 cassette into the bacmid genome. Transformants were selected on LB agar plates containing kanamycin (50 μg/mL), gentamicin (7 μg/mL), tetracycline (10 μg/mL), X-gal (40 μg/mL), and IPTG (100 μg/mL). White colonies were expanded, and bacmid DNA was isolated by alkaline lysis. Insertion of the VP2 gene was verified by PCR using M13 primers and sequencing. The bacmids were designated Bacmid-FPV-VP2 and Bacmidopt-FPV-VP2. The successful transposition of Tn7 and insertion of the VP2 gene were confirmed by blue-white screening and M13 PCR.

### 2.5. Cell Culture, Transfection, and Virus Amplification

Sf9 insect cells were maintained in Sf-900™ II SFM medium at 27 °C under standard suspension culture conditions. For recombinant baculovirus generation, Sf9 cells were seeded in six-well plates at a density of 8 × 10^5^ cells per well in 2.0 mL of fresh medium and allowed to equilibrate for 1 h prior to transfection. For each well, recombinant bacmid DNA (1.0 µg) was diluted in 100 µL of serum-free medium and gently mixed with 8 µL of Cellfectin™ II reagent pre-diluted in 100 µL of serum-free medium. The DNA–lipid complexes were incubated at room temperature for 20 min and then added dropwise to the cells. Transfected cells were incubated at 27 °C, and the transfection mixture was replaced with fresh medium after 5 h.

Transfected cultures were monitored daily for cytopathic effects (CPE) by light microscopy. At approximately 120 h post-transfection, the culture supernatant was harvested and clarified by centrifugation at 500× *g* for 10 min to remove cell debris. The clarified supernatant was collected as passage 0 (P0) viral stock and stored at 4 °C for short-term use (≤1 week). For virus amplification, mid-log-phase Sf9 cells were infected with P0 virus at a multiplicity of infection (MOI) of 0.1 in a total culture volume of 20 mL. After incubation at 27 °C for 96 h, the culture supernatant was harvested, clarified by low-speed centrifugation, and designated as the P1 viral stock. This amplification procedure was repeated sequentially under identical conditions to generate P2 and P3 viral stocks, which were used for subsequent protein expression, functional assays, and immunogenicity studies.

### 2.6. Analysis of Baculovirus Growth Kinetics and VP2 Protein Expression

Sf9 cells were infected with rBV-FPV-VP2 or rBVopt-FPV-VP2 at an MOI of 0.1 in six-well plates at 8 × 10^5^ cells per well. Cells were harvested every 12 h up to 96 h post-infection to assess growth kinetics, with cell viability determined by trypan blue exclusion and VP2 mRNA quantified by qPCR. Time points corresponding to peak VP2 transcript levels (typically observed around 72 h post-infection) were selected for downstream analyses, including infectious titer determination (TCID_50_), VP2 protein quantification by SDS-PAGE and Western blot, and total protein measurement using the BCA assay. Functional evaluation at these peak points included indirect immunofluorescence using anti-VP2 antibodies and Alexa Fluor–conjugated secondary antibodies, as well as hemagglutination assays using 1% porcine red blood cells. All experiments were performed in three independent biological replicates.

### 2.7. Optimization of Infection Conditions for VP2 Expression

Sf9 cells at a density of 2 × 10^6^ cells/mL in 50 mL of Sf-900™ II SFM medium were infected with rBV-FPV-VP2 or rBVopt-FPV-VP2 using MOIs of 0.01, 0.1, 1, and 3. Samples were collected every 12 h from 12 to 84 h post-infection, with cell density and viability determined by trypan blue exclusion and VP2 mRNA levels quantified by qPCR. All experiments were performed in three independent biological replicates. Time points corresponding to peak VP2 transcript levels (typically observed around 72 h post-infection) were selected for downstream analyses, including VP2 protein quantification by SDS-PAGE and Western blot and functional assessment via hemagglutination assay using 1% porcine red blood cells. The optimal infection conditions were determined based on integrated analysis of viral gene expression and VP2 protein functionality and were subsequently applied for large-scale VLP production under the identified MOI and harvest time.

### 2.8. Production and Purification of VP2 Virus-like Particles

Large-scale production of VP2 VLPs was performed under optimized infection conditions (MOI = 3; harvest at 72 h post-infection). Culture supernatants were clarified by centrifugation at 500× *g* for 10 min at 4 °C, then concentrated using a 100 kDa Macrosep Advance centrifugal device (Pall Corporation, Port Washington, NY, USA). The concentrated material was purified by size-exclusion chromatography (SEC) on a Superose 6 Increase 10/300 GL column pre-equilibrated with PBS, pH 7.4. Elution fractions containing VP2 were pooled and analyzed by SDS-PAGE. The self-assembly and structural morphology of the purified VLPs were confirmed by transmission electron microscopy (TEM) following negative staining.

### 2.9. Immunogenicity Evaluation and Challenge Study in Mice and Cats

The immunogenicity of purified FPV VLP was assessed in both murine and feline models. These experimental procedures were conducted at the animal facilities of Ludong University and were designed to evaluate the immunogenicity and protective efficacy of FPV VLPs in mice and cats.

In the mouse study, fifteen 6-week-old female BALB/c mice were randomly assigned to three groups (*n* = 5 per group): Group 1 received 10 μg of FPV VLPs formulated with MONTANIDE Gel 01 PR adjuvant; Group 2 received a commercially available inactivated vaccine administered according to the manufacturer’s recommended dosage and immunization schedule; and Group 3 received PBS as a negative control. Vaccinations were administered subcutaneously on day 0, followed by a booster on day 21. Experiments were conducted without blinding. Blood samples were collected on days 14, 21, 28, and 35, and FPV-specific IgG titers were determined using indirect ELISA.

In the cat study, nine 8-week-old SPF British Shorthair cats were randomly allocated into three groups (*n* = 3 per group): Group 1 received 50 μg of FPV VLPs with MONTANIDE Gel 01 PR adjuvant; Group 2 received a licensed commercial trivalent inactivated FPV vaccine, administered in strict accordance with the manufacturer’s recommended dosage and route of administration; and Group 3 received PBS. The vaccination schedule mirrored that of the mouse study, with primary immunization on day 0 and a booster on day 21. Experiments were conducted without blinding. Serum samples were collected on days 14, 21, 28, and 35 for assessment of FPV-specific IgG levels and hemagglutination inhibition (HI) activity.

For the feline challenge experiment, only the FPV VLP-immunized group and the PBS control group were included. SPF domestic cats (*n* = 3 per group) were orally challenged with 1 × 10^5^ TCID_50_ of the FPV072 strain 35 days after booster immunization under BSL-2 containment conditions. All experimental procedures were performed to minimize animal pain, suffering, and distress. Clinical parameters—including rectal temperature(Rectal temperature ≥ 39.5 °C was defined as fever), body weight, white blood cell (WBC) counts, and fecal consistency—were monitored daily throughout the 14-day post-challenge period. Rectal temperature and body weight were recorded every other day; WBC counts were assessed on days 0, 3, and 6 post-challenge; and fecal samples were collected every two days to evaluate viral shedding using a commercially available TaqMan probe-based real-time PCR kit specific for feline panleukopenia virus.

Animals were monitored daily for clinical signs, and humane endpoints were applied according to institutional guidelines. No unexpected adverse events were observed during the study. At 14 days post-challenge or upon reaching predefined humane endpoints, animals were humanely euthanized, and duodenal tissues were promptly collected for histopathological evaluation via hematoxylin-eosin (H&E) staining.

### 2.10. Statistical Analysis and Ethics Statement

Data are presented as mean ± SD. Statistical analyses were performed using GraphPad Prism 8.0. Comparisons between two groups were conducted using an unpaired two-tailed Student’s *t*-test, while comparisons involving two independent variables (e.g., time) were analyzed by two-way ANOVA followed by appropriate post hoc tests for pairwise comparisons. A *p*-value < 0.05 was considered statistically significant.

## 3. Results

### 3.1. Construction and Confirmation of Recombinant Bacmid and Virus Rescue

pFBD-FPV-VP2 and optimized pFBDopt-FPV-VP2 donor plasmids were assembled correctly. Colony PCR produced VP2-specific amplicons of the expected size, and restriction enzyme digestion generated fragment patterns consistent with theoretical predictions, confirming accurate plasmid construction (Figure S1). After transformation into DH10Bac cells, white-colony selection indicated successful Tn7 transposition, and M13 PCR verified precise insertion of the VP2 cassette into the bacmid genome (Figure S2).

Transfection of recombinant bacmids into Sf9 cells produced infectious baculoviruses. Cytopathic effects—cellular enlargement, granularity, and detachment—became evident at approximately 72 h post-transfection, indicating successful viral rescue (Figure S3). These results verify all upstream genetic steps and establish a solid basis for subsequent analyses of replication kinetics and VP2 expression.

### 3.2. VP2 Expression Kinetics and Yield at the Peak Production Time Point

**Figure 2:**
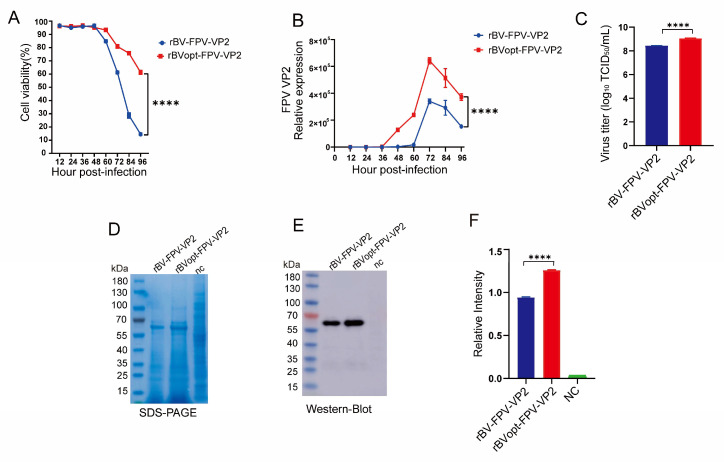
Analysis of rBVopt-FPV-VP2 infection and VP2 expression in Sf9 cells. (**A**) Cell viability during infection (*n* = 3). (**B**) Viral genome replication by qPCR (*n* = 3). (**C**) Infectious titers by TCID_50_ at 72 hpi (*n* = 3). (**D**) SDS-PAGE of total cellular proteins. (**E**) Western blot of VP2 expression. (**F**) Densitometric analysis of VP2 signals (*n* = 3). Data are presented as mean ± SD from independent samples. Statistical comparisons for panels A and B were performed using two-way ANOVA followed by post hoc multiple-comparison tests, while comparisons for panels (**C**,**F**) were performed using unpaired two-tailed Student’s *t*-test. Statistical significance is indicated as **** *p* < 0.0001.

Sf9 cells infected with rBVopt-FPV-VP2 exhibited improved infection dynamics compared with those infected with rBV-FPV-VP2. Cell viability remained consistently higher in the optimized group (~80% vs. ~60% at 72 hpi; Figure 2A), indicating delayed apoptosis. Viral genome copy numbers peaked at 72 hpi, with the optimized virus reaching approximately 1.5-fold higher levels (Figure 2B), accompanied by a marked increase in infectious titers (1.09 ± 0.12 × 10^9^ vs. 2.82 ± 0.35 × 10^8^ TCID_50_/mL; Figure 2C).

VP2 protein expression was correspondingly enhanced, as evidenced by stronger bands in SDS-PAGE and Western blot analyses (33% increase; Figure 2D–F). Consistent with these findings, BCA analysis revealed a higher overall total protein yield in the optimized group (256.3 ± 8 mg/L vs. 174.3 ± 4.5 mg/L; Table 1), representing a 1.47-fold increase compared with the conventional BEVS control

Collectively, these results demonstrate that rBVopt-FPV-VP2 significantly enhances recombinant virus replication and VP2 production at the peak production time point, providing a quantitative basis for subsequent functional characterization.

### 3.3. Functional Characterization of VP2 at the Peak Production Time Point

**Figure 3:**
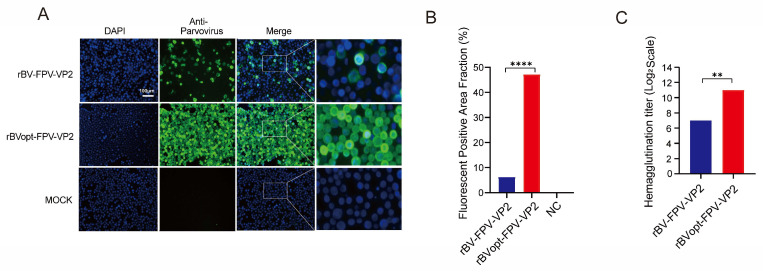
VP2 expression and biological activity post-infection. (**A**) Indirect immunofluorescence of Sf9 cells (green: VP2; blue: nuclei; scale bar = 100 μm). (**B**) Fluorescence area ratio; rBVopt-FPV-VP2 shows >7-fold higher signal (*n* = 3). (**C**) Hemagglutination titers; optimized virus exhibits higher activity (*n* = 3). Data are presented as mean ± SD from independent experiments. Statistical comparisons for panels (**B**,**C**) were performed using an unpaired two-tailed Student’s *t*-test. Statistical significance is indicated as ** *p* < 0.01 and **** *p* < 0.0001.

We then evaluated the functional activity of VP2. Indirect immunofluorescence at 72 hpi confirmed VP2 expression in Sf9 cells for both constructs, with no signal in uninfected controls (Figure 3A). The optimized virus exhibited stronger fluorescence and a higher proportion of positive cells (fluorescence area ratio: 47.177 vs. 6.236; Figure 3B). Hemagglutination assays showed higher HA titers for rBVopt-FPV-VP2 (2^11^ vs. 2^7^; Figure 3C), indicating increased production of biologically active VP2.

These observations confirm that rBVopt-FPV-VP2 produces VP2 with enhanced biological activity, demonstrating improved functional quality of the expressed protein rather than increased quantity.

### 3.4. Optimization of Infection Conditions for FPV VP2 Expression

**Figure 4:**
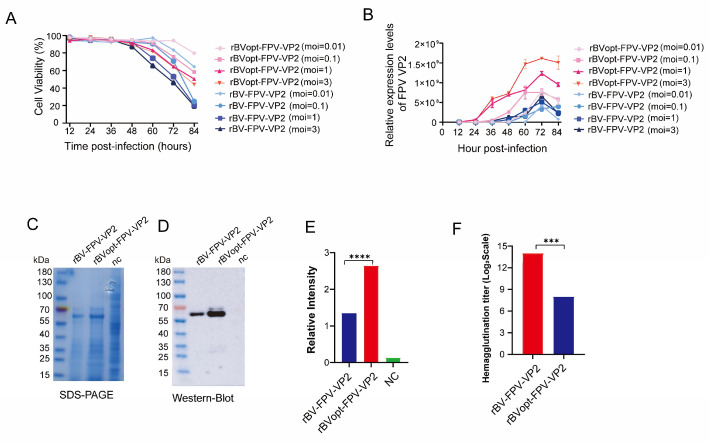
Optimization of FPV VP2 expression in Sf9 cells. (**A**) Cell viability monitored under different MOIs (*n* = 3). (**B**) Viral genome replication kinetics across MOIs (*n* = 3). (**C**) SDS-PAGE analysis of VP2 expression. (**D**) Western blot detection of VP2. (**E**) Densitometric quantification of VP2 bands (*n* = 3). (**F**) Hemagglutination titers under optimized infection conditions (*n* = 3). Data are presented as mean ± SD from independent experiments. Statistical comparisons for panels (**E**,**F**) were performed using an unpaired two-tailed Student’s *t*-test. Statistical significance is indicated as *** *p* < 0.01 and **** *p* < 0.0001.

To determine the optimal production parameters, Sf9 cells were infected at MOIs of 0.01, 0.1, 1, and 3. The optimized virus, rBVopt-FPV-VP2, maintained higher cell viability throughout infection (Figure 4A). Viral genome replication peaked at 72 hpi across all MOIs, with the optimized virus exhibiting higher copy numbers and prolonged active replication (Figure 4B).

Based on these results, an MOI of 3 and harvest at 72 h were selected for maximal VP2 yield. Under these conditions, SDS-PAGE and Western blot analyses confirmed further enhancement of VP2 expression, and hemagglutination titers reached 2^14^, indicating improved VLP assembly (Figure 4C–F).

These findings demonstrate that the optimized infection conditions further enhance VP2 production and promote functional VLP formation, supporting downstream VLP characterization.

### 3.5. Production and Structural Characterization of Virus-like Particles

**Figure 5:**
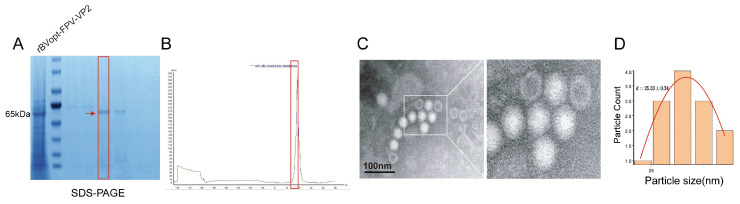
Production and structural characterization of FPV VP2 virus-like particles (VLPs). (**A**) SDS-PAGE analysis of the purified VP2 protein; the red box marks the target band corresponding to the sample used for SEC analysis. (**B**) Size-exclusion chromatography (SEC) UV absorbance profile of the same purified sample (matching the red-boxed lane in panel (**A**)). (**C**) Transmission electron microscopy (TEM) images of negatively stained VP2 VLPs showing uniform icosahedral particles (scale bar = 100 nm). (**D**) Particle size distribution of purified VP2 VLPs.

Large-scale expression of FPV VP2 under optimized conditions (MOI = 3, harvest at 72 h) yielded abundant VLPs. Size-exclusion chromatography (SEC) effectively resolved high-molecular-weight VP2 assemblies from contaminating species, with SDS-PAGE analysis confirming a single band at approximately 65 kDa and minimal detectable impurities (Figure 5A,B). TEM revealed uniformly spherical particles with a diameter of approximately 25 nm, closely resembling the morphology of native feline parvovirus capsids. Particle size distribution analysis further demonstrated a narrow size range, supporting the structural homogeneity of the VLP preparation (Figure 5C,D).

These results indicate that the optimized BEVS facilitates correct VP2 folding and efficient self-assembly into structurally authentic virus-like particles.

### 3.6. Immunogenicity of FPV VLPs in Mouse and Feline Models

**Figure 6:**
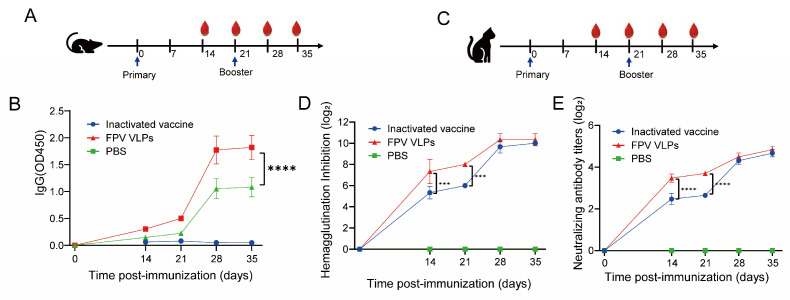
Immunogenicity of FPV VLPs in mice and cats. (**A**) Immunization schedule in mice; (**B**) Immunization schedule in cats; (**C**) Dynamic FPV-specific IgG antibody responses in mice; (**D**) Hemagglutination inhibition (HI) titers in cats; (**E**) Neutralizing antibody titers in cats. Data are presented as mean ± SD. Statistical comparisons between groups at each time point were performed using two-way ANOVA followed by post hoc multiple-comparison tests. Statistical significance is indicated as *** *p* < 0.001 **** *p* < 0.0001. Each group included *n* = 5 mice or *n* = 3 cats.

FPV VLPs elicited rapid and robust humoral immunity in both murine and feline models. In mice, FPV-specific IgG antibodies were detectable 14 days after the primary immunization and increased further after the booster, reaching stable peak levels by day 28 (Figure 6A,B). No appreciable antibody response was observed in the PBS control group.

In cats, FPV VLPs induced early HI and neutralizing antibody responses, with detectable titers 14 days after the first immunization (HI 2^8^; neutralizing 1:37) and further increases following the booster dose (Figure 6C–E). The rapid induction of high antibody titers demonstrates that FPV VLPs can provide early, strong, and effective humoral protection. This early immune advantage is particularly relevant for kittens and highlights the potential application of FPV VLPs in urgent immunization scenarios.

Overall, these results indicate that FPV VLPs are highly immunogenic and capable of eliciting fast and effective antibody responses, supporting their development as a novel FPV vaccine candidate.

### 3.7. Protective Efficacy of FPV VLPs Following Virulent Challenge

**Figure 7:**
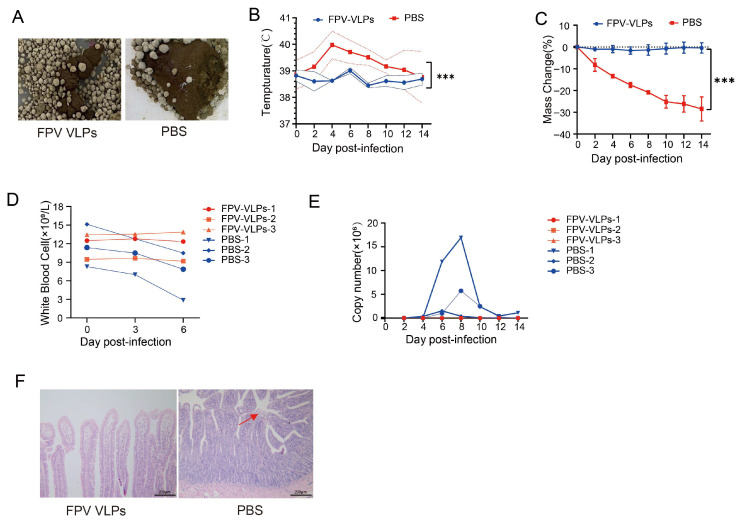
Protective efficacy of FPV VLP immunization following virulent challenge. (**A**) Clinical diarrhea scores. (**B**) Rectal temperature dynamics. (**C**) Body weight changes. (**D**) Peripheral white blood cell (WBC) counts. (**E**) Fecal viral shedding. (**F**) Representative duodenal histopathology at 14 days post-challenge (scale bar = 200 μm). Red arrow indicates areas of epithelial damage and villous atrophy in the control group. Control group (*n* = 3), VLP-vaccinated group (*n* = 3). Control group (*n* = 3) and VLP-vaccinated group (*n* = 3). Data are presented as mean ± SD. Statistical comparisons for panels (**B**,**C**) were performed using two-way ANOVA followed by post hoc multiple-comparison tests. Statistical significance is indicated as *** *p* < 0.001.

Twenty-eight days after booster immunization, cats immunized with FPV VLP or PBS were orally challenged with the virulent FPV072 strain. Clinical signs were observed exclusively in the control group and included severe diarrhea (Figure 7A), whereas all VLP-vaccinated cats remained clinically normal throughout the study. Rectal temperatures in the control group peaked at approximately 4 days post-challenge, with one cat reaching 40.5 °C, while VLP-immunized cats maintained stable physiological temperatures (~38.5 °C) for the duration of the observation period (Figure 7B). By day 10 post-infection, control animals exhibited an 8–10% reduction in body weight, in contrast to minimal fluctuations (±2%) in the VLP group (Figure 7C). Peripheral white blood cell counts in the control group declined by approximately 50% by 3 days post-infection, consistent with FPV-induced leukopenia, whereas vaccinated cats maintained normal hematological values (Figure 7D).

The VLP vaccine potently suppressed viral replication and prevented pathological damage in cats following virulent challenge. Fecal viral loads in vaccinated animals remained consistently below detectable levels throughout the observation period, whereas control cats exhibited peak viral titers of approximately 10^7^ copies/g (Figure 7E). Correspondingly, intestinal tissues from vaccinated cats preserved intact mucosal architecture with minimal histopathological changes, while control animals displayed severe villous atrophy and extensive epithelial destruction (Figure 7F).

Collectively, these findings demonstrate that FPV VLP immunization provided robust clinical and virological protection in the study cohort, effectively preventing fever, weight loss, leukopenia, intestinal pathology, and high-level viral shedding following challenge.

## 4. Discussion

This study developed a temporally coordinated optimized BEVS, integrating the hr1-p6.9-p10 composite regulatory cassette and the Ac-ie-01 anti-apoptotic gene, and for the first time systematically evaluated this optimized BEVS for FPV VP2 expression and VLP production [10]. The optimized BEVS substantially enhanced recombinant virus replication and VP2 protein expression, supporting robust assembly of structurally uniform and morphologically intact VLPs. These VLPs elicited rapid and potent humoral immune responses in both mice and cats, with strong clinical and virological protection within the tested feline cohort, while acknowledging that the small sample size (*n* = 3 per group) limits statistical power and generalizability.

At the mechanistic level, the composite regulatory cassette significantly extended the transcriptional window of VP2 by enabling early promoter activation and sustained expression during the late phase of infection [21,22]. Concurrently, Ac-ie-01 expression delayed apoptosis in Sf9 cells, preserving cellular metabolic activity and prolonging the viral replication cycle [23]. Experimental validation confirmed these design principles: the optimized recombinant virus showed a ~1.5-fold increase in viral genome copy number at 72 hpi, a 3.9-fold enhancement in infectious titer, and a 33% increase in VP2 protein expression, with a 1.47-fold rise in total protein yield. Notably, HA activity increased dramatically from 2^7^ to 2^11^, demonstrating that the optimized BEVS not only boosted antigen quantity but also substantially improved the yield of functionally active VP2.

Purified VLPs displayed uniform particle size (~25 nm) and structural morphology closely resembling native FPV virions, confirming that the optimized BEVS effectively supports efficient assembly of complex viral capsid proteins. Although total VP2 yield increased moderately (1.47-fold), such incremental improvements are typical for structurally complex VLPs produced using BEVS, where protein expression and particle assembly are constrained by host cell capacity. In this context, the observed enhancement represents a meaningful platform-level improvement achieved without compromising VLP assembly efficiency, structural integrity, or functional activity [23,24].

Immunogenicity evaluations in mice and cats further demonstrate the distinct advantages of the VLP-based vaccine. FPV VLPs induced rapid and robust humoral immune responses, as reflected by accelerated antibody kinetics after immunization. These observations suggest that the particulate structure of VLPs enhances antigen uptake and promotes efficient B-cell activation, leading to timely antibody-mediated immunity [25,26]. However, early-time-point challenge experiments or mechanistic analyses of innate and cellular immune responses were not included. Consequently, any inference regarding rapid-onset protection is based on antibody kinetics rather than direct experimental demonstration. Future studies with earlier challenges and broader immunological assessments will be necessary to definitively evaluate accelerated protective efficacy.

The challenge experiment provided functional validation of the protective efficacy of the VLP vaccine. Vaccinated cats exhibited stable clinical parameters, including body temperature, body weight, and white blood cell counts, and no viral shedding was detected in fecal samples. Histopathological analysis revealed well-preserved intestinal villi with minimal inflammatory infiltration. In contrast, control cats developed hallmark signs of FPV infection, including fever, weight loss, leukopenia, and extensive tissue damage. Given the limited cohort size, these outcomes should be interpreted cautiously, indicating strong protection within the tested cohort but requiring confirmation in larger, independent cohorts.

Although this study demonstrates the substantial impact of the optimized BEVS platform on FPV VP2 expression and vaccine development, several limitations warrant further investigation. First, the feline challenge experiment involved a small number of animals, limiting statistical power; therefore, the protective outcomes observed here should be regarded as preliminary. Second, while humoral immunity was robustly characterized, FPV-specific cellular immune responses were not systematically assessed. Observed protective efficacy is inferred primarily from antibody responses rather than demonstrated experimentally. In addition, formal stability testing of the purified FPV VP2 VLPs was not conducted. VLP preparations were freshly purified prior to each experiment and verified by SDS-PAGE and TEM to ensure structural integrity; however, these measures do not replace systematic stability assessment.

Future studies incorporating expanded animal cohorts and more comprehensive immunological analyses—including early challenge experiments, innate immune activation, and cellular immunity—together with rigorous evaluation of VLP stability under different storage conditions and over extended periods, will be essential to fully define the protective mechanisms, durability, shelf-life, and translational potential of FPV VLP vaccination. Furthermore, the generalizability of the optimized BEVS to other structurally complex viral antigens, particularly those forming icosahedral capsids, remains to be explored and warrants systematic evaluation for broader applicability in subunit vaccine development [27,28].

In summary, this study establishes a robust and scalable strategy for the efficient production of FPV VLPs, demonstrating that the optimized BEVS significantly enhances recombinant protein expression, improves functional antigen yield, and potentiates vaccine-induced immune responses. This optimized BEVS-based engineering approach provides a valuable framework for the development of VLP-based veterinary vaccines, holds promise for human vaccine applications, and offers a solution to key bottlenecks in large-scale antigen manufacturing using BEVS, thereby advancing its utility in industrial vaccine production.

## 5. Conclusions

In summary, this study demonstrates that FPV VLPs can be efficiently produced using an optimized BEVS and exhibit robust immunogenicity in both mice and cats. Notably, FPV VLPs induce comparably earlier and stronger antibody responses compared to a commercial inactivated vaccine, highlighting their intrinsic advantages as particulate antigens. These findings provide a solid experimental foundation for advancing FPV VLPs into practical vaccine development and support their potential as a next-generation prophylactic strategy for protecting young kittens against FPV infection.

## Figures and Tables

**Table 1:** Protein Quantitative Detection.

Protein Name	Total Protein Content (mg/L)
rBV-FPV-VP2	174.3 ± 4.5 mg/L
rBVopt-FPV-VP2	256.3 ± 8 mg/L

## Data Availability

The original contributions presented in this study are included in the article. Further inquiries can be directed to the corresponding authors.

## Supplementary Materials

The following supporting information can be downloaded at: https://www.mdpi.com/article/10.3390/vaccines14010077/s1, Figure S1: Verification of recombinant donor plasmids. Figure S2: PCR confirmation of recombinant bacmids. Figure S3. Cytopathic effects (CPE) induced by recombinant baculoviruses in Sf9 cells.

## References

[1] MSD Veterinary Manual Feline Panleukopenia [EB/OL]. https://www.msdvetmanual.com/digestive-system/infectious-diseases-of-the-gastrointestinal-tract-in-small-animals/feline-panleukopenia.

[2] Jacobson L.S., Janke K.J., Giacinti J., Weese J.S. (2021). Diagnostic testing for feline panleukopenia in a shelter setting: A prospective, observational study. J. Feline Med. Surg..

[3] Wen Y., Tang Z., Wang K., Geng Z., Yang S., Guo J., Chen Y., Wang J., Fan Z., Chen P. (2024). Epidemiological and molecular investigation of feline panleukopenia virus infection in China. Viruses.

[4] Liu C., Si F., Li H., Gao J., Sun F., Liu H., Yi J. (2023). Identification and genome characterization of novel feline parvovirus strains isolated in Shanghai, China. Curr. Issues Mol. Biol..

[5] Truyen U., Parrish C.R. (2013). Feline panleukopenia virus: Its interesting evolution and current problems in immunoprophylaxis against a serious pathogen. Vet. Microbiol..

[6] Wang T., Wu H., Wang Y., Guan Y., Cao Y., Wang L., Wang M., Tan F., Pang W., Tian K. (2025). Virus-like particle vaccine for feline panleukopenia: Immunogenicity and protective efficacy in cats. Vaccines.

[7] Chang D., Liu Y., Chen Y., Hu X., Burov A., Puzyr A., Bondar V., Yao L. (2020). Study of the immunogenicity of the vp2 protein of canine parvovirus produced using an improved baculovirus expression system. BMC Vet. Res..

[8] Li J., Zeng Y., Li L., Peng J., Yan Q., Ye Z., Zhang Y., Li W., Cao L., Zhou D. (2024). Development of a recombinant lactobacillus plantarum oral vaccine expressing vp2 protein for preventing feline panleukopenia virus. Vet. Microbiol..

[9] Zhou H., Yao G., Cui S. (2010). Production and purification of vp2 protein of porcine parvovirus expressed in an insect-baculovirus cell system. Virol. J..

[10] Huang N., Wei Y., Li J. (2025). Insect cell expression system: Advances in applications, engineering strategies, and bioprocess development. J. Biol. Eng..

[11] Irons S.L., Chambers A.C., Lissina O., King L.A., Possee R.D. (2018). Protein production using the baculovirus expression system. Curr. Protoc. Protein Sci..

[12] Van Oers M.M., Pijlman G.P., Vlak J.M. (2015). Thirty years of baculovirus–insect cell protein expression: From dark horse to mainstream technology. J. Gen. Virol..

[13] Xia L., Luo G., Wu M., Wang L., Zhang N., Wu C., Yin Y. (2021). Self-assembled raccoon dog parvovirus vp2 protein confers immunity against rdpv disease in raccoon dogs: In vitro and in vivo studies. Virol. J..

[14] Fabre M.L., Arrías P.N., Masson T., Pidre M.L., Romanowski V. (2020). Baculovirus-derived vectors for immunization and therapeutic applications. Emerging and Reemerging Viral Pathogens.

[15] Feng E., Luo G., Wang C., Liu W., Yan R., Bai X., Cheng Y. (2025). Generation and immunogenicity of virus-like particles based on the capsid protein of a chinese epidemic strain of feline panleukopenia virus. Vet. Sci..

[16] Grose C., Putman Z., Esposito D. (2021). A review of alternative promoters for optimal recombinant protein expression in baculovirus-infected insect cells. Protein Expr. Purif..

[17] Steele K.H., Stone B.J., Franklin K.M., Fath-Goodin A., Zhang X., Jiang H., Webb B.A., Geisler C. (2017). Improving the baculovirus expression vector system with vankyrin-enhanced technology. Biotechnol. Prog..

[18] Chen Z., Li C., Zhu Y., Wang B., Meng C., Liu G. (2012). Immunogenicity of virus-like particles containing modified goose parvovirus vp2 protein. Virus Res..

[19] Gomez-Sebastian S., Lopez-Vidal J., Escribano J.M. (2014). Significant productivity improvement of the baculovirus expression vector system by engineering a novel expression cassette. PLoS ONE.

[20] Xie Q., Sun Z., Xue X., Pan Y., Zhen S., Liu Y., Zhan J., Jiang L., Zhang J., Zhu H. (2024). China-origin g1 group isolate fpv072 exhibits higher infectivity and pathogenicity than g2 group isolate fpv027. Front. Vet. Sci..

[21] Hong M., Li T., Xue W., Zhang S., Cui L., Wang H., Zhang Y., Zhou L., Gu Y., Xia N. (2022). Genetic engineering of baculovirus-insect cell system to improve protein production. Front. Bioeng. Biotechnol..

[22] Hu Y.C. (2005). Baculovirus as a highly efficient expression vector in insect and mammalian cells. Acta Pharmacol. Sin..

[23] Lopez-Vidal J., Gomez-Sebastian S., Barcena J., Nuñez M.D.C., Martínez-Alonso D., Dudognon B., Guijarro E., Escribano J.M. (2015). Improved production efficiency of virus-like particles by the baculovirus expression vector system. PLoS ONE.

[24] Choi J.-B., Lee J.-H., Kim E.-H., Kim J.-D., Kim S.-Y., Oh J.-M., Woo S.-D., Kim H., Han B.-K. (2025). Productivity improvement of human papillomavirus-like particles in insect cells using hyper-expression baculovirus vector. Vaccines.

[25] Lua L.H., Connors N.K., Sainsbury F., Chuan Y.P., Wibowo N., Middelberg A.P.J. (2014). Bioengineering virus-like particles as vaccines. Biotechnol. Bioeng..

[26] Roldão A., Mellado M.C., Castilho L.R., Carrondo M.J., Alves P.M. (2010). Virus-like particles in vaccine development. Expert Rev. Vaccines.

[27] Bruder M.R., Aucoin M.G. (2023). Evaluation of virus-free manufacture of recombinant proteins using crispr-mediated gene disruption in baculovirus-infected insect cells. Vaccines.

[28] Hong Q., Liu J., Wei Y., Wei X. (2023). Application of baculovirus expression vector system (bevs) in vaccine development. Vaccines.

