# Supplementary material for: Donor Plasmid Optimization Enhances Expression of Feline Parvovirus VP2 Protein in the Baculovirus Expression Vector System
Source: Vaccines (Basel). 2026 Jan 10;14(1):77. doi: 10.3390/vaccines14010077 (PMC12846515; doi:10.3390/vaccines14010077)
Supplement: Supplementary file 1 [file vaccines-14-00077-s001.zip › vaccines-4070634-supplementary.pdf]

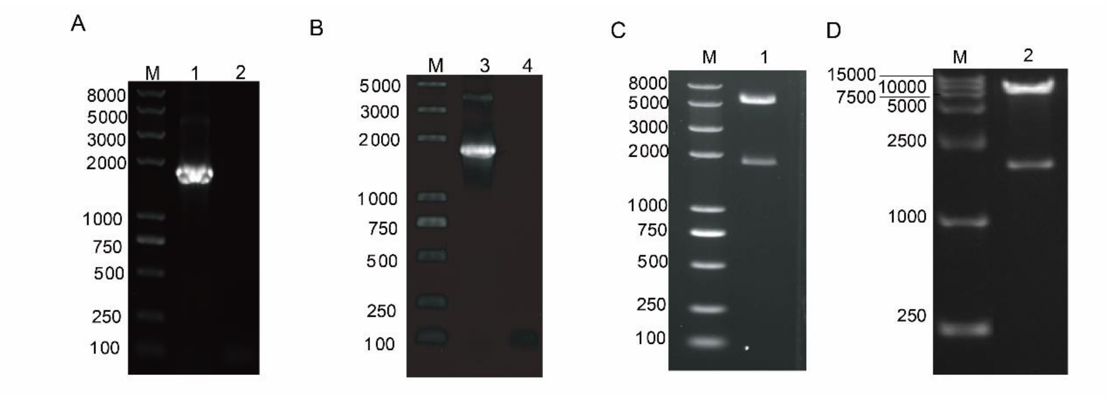

Figure S1. Verification of recombinant donor plasmids. (A) PCR identification of pFBD-FPV-VP2 showing the VP2-specific 1755 bp band. (B) PCR identification of pFBDopt-FPV-VP2 showing the VP2-specific 1755 bp band.(C) BamHI/HindIII digestion of pFBD-FPV-VP2 releasing the expected 1755 bp insert and 5238 bp backbone.(D) KpnI/XhoI digestion of pFBDopt-FPV-VP2 yielding the expected 1755 bp insert and 11823 bp vector fragment.

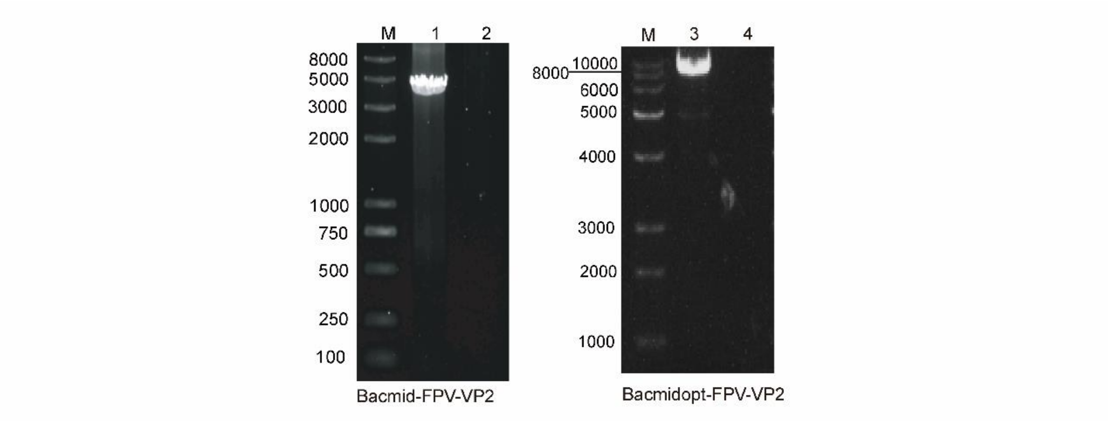

Figure S2. PCR confirmation of recombinant bacmids. (A) PCR verification of Bacmid-FPV-VP2. Lane M: DNA marker; Lane 1: positive amplification of the VP2 insert; Lane 2: negative control. (B) PCR verification of Bacmidopt-FPV-VP2. Lane M: DNA marker; Lane 3: positive amplification of the optimized VP2 insert; Lane 4: negative control.

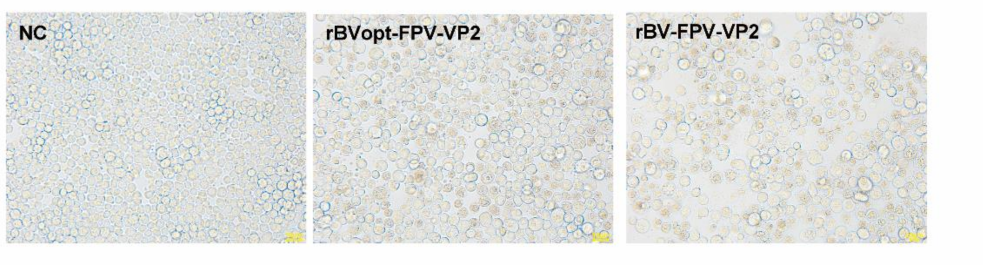

Figure S3. Cytopathic effects (CPE) induced by recombinant baculoviruses in Sf9 cells. Morphological changes in Sf9 cells infected with rBVopt-FPV-VP2 or rBV-FPV-VP2 were evaluated at 72 hours post-infection. Scale bar = 10 μm.
